# Studying and Analyzing Humane Endpoints in the Fructose-Fed and Streptozotocin-Injected Rat Model of Diabetes

**DOI:** 10.3390/ani13081397

**Published:** 2023-04-18

**Authors:** Rita Silva-Reis, Ana I. Faustino-Rocha, Jéssica Silva, Abigaël Valada, Tiago Azevedo, Lara Anjos, Lio Gonçalves, Maria de Lurdes Pinto, Rita Ferreira, Artur M. S. Silva, Susana M. Cardoso, Paula A. Oliveira

**Affiliations:** 1 LAQV-REQUIMTE, Department of Chemistry, University of Aveiro, 3810-193 Aveiro, Portugal; 2 CITAB Inov4Agro—Institute for Innovation, Capacity Building and Sustainability of Agri-Food Production, University of Trás-Os-Montes and Alto Douro, 5000-801 Vila Real, Portugal; 3 Department of Zootechnics, School of Sciences and Technology, University of Évora, 7000-812 Évora, Portugal; 4 Comprehensive Health Research Center, 7000-812 Évora, Portugal; 5 Engineering Department, University of Trás-Os-Montes and Alto Douro, 5000-801 Vila Real, Portugal; 6 Institute for Systems and Computer Engineering, Technology and Science, 4200-465 Porto, Portugal; 7 Animal and Veterinary Research Center (CECAV), University of Trás-Os-Montes and Alto Douro, 5000-801 Vila Real, Portugal; 8 Department of Veterinary Sciences, University of Trás-Os-Montes and Alto Douro, 5000-801 Vila Real, Portugal

**Keywords:** rat model, welfare, hyperglycemia, polyuria, polyphagia, polydipsia

## Abstract

**Simple Summary:**

This study assessed a humane endpoint scoring system to detect animal suffering in a rat model of type 2 diabetes. Sprague-Dawley male rats were divided into control and induced (fructose-fed and streptozotocin (STZ) administration) groups. Induced animals drank 10% fructose for 14 days, then received STZ (40 mg/kg) intraperitoneally. Weekly monitoring of body weight, water, and food consumption, 14 parameters of animal welfare, and blood glucose levels were conducted. Results showed weight loss, polyuria, polyphagia, and polydipsia, as well as lack of grooming, narrowing of the orbital area, curved posture, liquid/pasty diarrhea, and abdominal distension. The most useful parameters to evaluate humane endpoints in this type 2 diabetes rat induction model were dehydration, absence of grooming, the posture of the animals, abdominal visualization and palpation, and fecal appearance. The glycemia was significantly higher in the induced group, validating the animal model of diabetes. The humane endpoints table was suitable for monitoring animal welfare.

**Abstract:**

This work aimed to define a humane endpoint scoring system able to objectively identify signs of animal suffering in a rat model of type 2 diabetes. Sprague-Dawley male rats were divided into control and induced group. The induced animals drink a 10% fructose solution for 14 days. Then, received an administration of streptozotocin (40 mg/kg). Animals’ body weight, water and food consumption were recorded weekly. To evaluate animal welfare, a score sheet with 14 parameters was employed. Blood glucose levels were measured at three time points. After seven weeks of initiating the protocol, the rats were euthanized. The induced animals showed weight loss, polyuria, polyphagia, and polydipsia. According to our humane endpoints table, changes in animal welfare became noticeable after the STZ administration. None of the animals hit the critical score limit (four). Data showed that the most effective parameters to assess welfare in this type 2 diabetes rat induction model were dehydration, grooming, posture, abdominal visualization, and stool appearance. The glycemia was significantly higher in the induced group when compared to the controls (*p* < 0.01). Induced animals’ murinometric and nutritional parameters were significantly lower than the controls (*p* < 0.01). Our findings suggest that in this rat model of type 2 diabetes with STZ-induced following fructose consumption, our list of humane endpoints is suitable for monitoring the animals’ welfare.

## 1. Introduction

Several animal models have been used to better understand the biopathology of chronic diseases, such as diabetes [1]. The use of laboratory animals raises many ethical concerns, particularly when stress and pain are induced in the animals. Humane endpoints have become a major element in experiments involving laboratory animals to overdue these ethical issues, applying the 3R’s principles (Replacement, Reduction and Refinement) [2]. According to the Canadian Council on Animal Care (CCAC), humane endpoints are physiological or behavioral signs that define the critical stage at which the animal’s pain and/or stress must be finished or mitigated through decisions such as no application of a painful procedure, treatment to alleviate pain and/or distress, or ultimately the animals’ sacrifice [3]. The assessment of the animals’ pain and stress can become subjective, and the researchers may incorrectly interpret certain signs as indicators of pain even when they are not. To reduce misinterpretations, scoring systems have been developed, thus allowing the assessment of animal welfare through the attribution of a score to each parameter. It is worth noting that the parameters examined in a certain study must be adjusted to the study’s goal. Normally, at the end of the evaluation, the total score of each animal is recorded and determines whether an animal should or not be removed from the protocols [4,5,6]. The evolution of chromodacryorrhea [7], the animals’ grooming habits (self-grooming) [8], the Rat Grimace scale (which allows the assessment of pain and stress in animals through an analysis of the animals’ expressions) [9], body weight [10], and body temperature using infrared thermometers [11] are some of the parameters generally used to define humane endpoints in animal models of disease. However, a systematic approach published by David Morton [4] suggested additional parameters that should be included in the evaluation of humane endpoints in a rat model of type 2 diabetes, the most prevalent type of diabetes worldwide [12]. This study utilized a non-genetic animal model that mimics the pathogenesis of type 2 diabetes by administering STZ to animals that had been fed with fructose in their drinking water [13]. Various parameters were compiled to assess the welfare of animals throughout experimental trials, incorporating those proposed by David Morton [12] and additional ones, such as signs of dehydration, diarrhea, abdominal distension, and posture. Moreover, this work aimed to clearly define a humane endpoint scoring system able to objectively identify signs of animal suffering before the development of severe disease in this model of type 2 diabetes, mitigating all ethical and animal welfare concerns.

## 2. Materials and Methods

### 2.1. Ethical Statements

The experimental protocol was approved by the University of Trás-os-Montes and Alto Douro Ethics Committee (“ORBEA—Órgão Responsável pelo Bem-Estar e Ética Animal; approval no 852-e-CITAB-2020 and A_1-e-CITAB-2021). All the experiments performed on the animals were carried out under the European (European Directive 2010/63/EU) and national legislation (Decree-Law no. 113/2013) on the protection of animals used for scientific purposes.

### 2.2. Animals and Chemicals

Twenty-four Sprague-Dawley male rats (*Rattus norvegicus*) at three weeks of age and weighing 134.24 ± 10.11 g were obtained from Envigo (Barcelona, Spain). The animals were kept in polycarbonate cages with smooth surfaces and rounded edges (1500 U Eurostandard Type IV S, Tecniplast, Buguggiate, Italy), three to five animals per cage, with appropriate identification. Animals were negative for viruses, bacteria, mycoplasma, fungi, parasites, and pathological lesions. The bedding for the animals was prepared from corncob (Ultragene, Santa Comba Dão, Portugal), and it was changed every week. Following the introduction of fructose and the administration of STZ, the animals urinate more frequently, demanding daily bedding changes. Polyvinyl chloride tubes were placed in each cage to promote the rat’s environmental enrichment. With these materials, animals can hide and mimic a natural environment. This research was conducted in a controlled environment with a 12-h light/12-h dark cycle at a temperature of 20 ± 2 °C and relative humidity of 50 ± 10%. Fructose, acquired from BioPortugal-Químico, Farmacêutica, Lda (Porto, Portugal), was prepared daily in the drinking water at a concentration of 10% immediately before being provided to the animals to avoid fermenting and placed in opaque bottles. STZ was obtained from BioPortugal-Químico, Farmacêutica, Lda (Porto, Portugal) and prepared before its use in a 0.1 M citrate buffer solution (pH = 4.3).

### 2.3. Experimental Protocol

**Figure 1:**
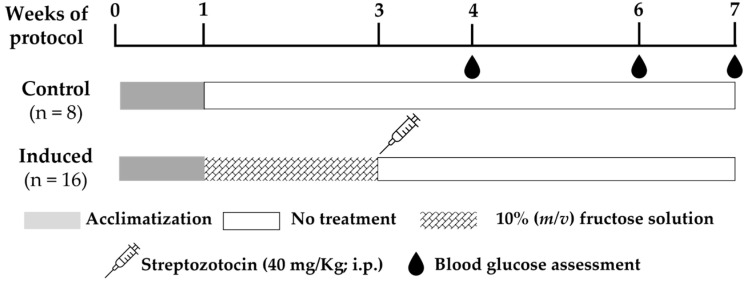
Experimental design.

After one week of acclimatization, the rats were randomly assigned to two experimental groups: group control (n = 8) and group induced (n = 16). The experimental design was done without “nuisances”, with no need for randomized block designs. After reviewing various published papers concerning animal models of diabetes [14,15,16], we identified a mortality rate between 20–40% and a rate of disease induction from 58% to 70%. So, to ensure the necessary sampling to have a statistical analysis power of the results, the number of animals differs between groups. This number intends to be conservative and not compromise the analysis of the results, considering Russell and Burch’s 3R principles. Thus, the number of animals per group was calculated by the Experimental Design Assistant (https://efa.nc3rs.org.uk (accessed on 1 May 2021)). A higher number of animals was used in the group induced due to the higher mortality rate expected in the animals with diabetes when compared with those animals from group control [13,14,15,16]. At 4 weeks of age (beginning of week 1), the animals from the induced group began to drink a 10% fructose solution prepared as indicated above for 14 consecutive days (two weeks). After this, all animals from both groups were exposed to a 12 h overnight fast. Then, the animals from the group induced received a single intraperitoneal administration of STZ (40 mg/kg), while the animals from group control received an injection of the vehicle (0.1 M citrate buffer). Throughout the trial, the animals had unlimited access to tap water via capped bottles and a standard laboratory diet (SAFE^®^ Custom Diets, Augy, France). Only the bottles with the 10% (*m/v*) fructose solution were available to the induced animals during fructose administration (Figure 1).

### 2.4. Body Weight, Food, and Water Consumption

Weekly, individual rats’ weight, as well as food and water consumption, were determined, using a top-loading scale (Mettler PM4000, LabWrench, Midland, ON, Canada).

The body weight gain (BWG) for each rat (%) was also determined using the following formula [17]:

(1) $$BWG=\frac{Body\;weight\;at\;a\;certain\;point-Initial\;body\;weight}{Body\;weight\;at\;a\;certain\;point}\times 100,$$

The following formula was used to calculate the mean daily food consumption for each rat (g) [10]:

(2) $$Daily\;food\;consumption=\frac{Food\;weight\;(beggining\;of\;the\;week)-Food\;weight\;(end\;of\;the\;week)}{No.\;of\;animals\;in\;the\;cage\times No.\;of\;days}$$

Initially, water consumption was determined similarly to food consumption. However, with the disease development, the animals from the group induced started to drink more water and, together with the weekly water losses, it became difficult to assess the real water consumption. In this way, the research team decided to calculate the water consumption for just 24 h, with three bottles available per cage. Thus, water consumption from week 4 onwards was calculated by dividing the total cage water consumption by the number of animals in the cage.

### 2.5. Scoring Sheet

To monitor the animals’ welfare, the registration of humane endpoints was implemented during the experimental protocol, according to Table 1. The parameters were defined in accordance with CCAC guidelines [3] and updated based on earlier works performed by our team [10,17,18]. A score from zero to three was given for each parameter listed in the table. The animals were observed daily, and the scores were recorded once a week by three independent observers to avoid bias. The data were recorded independently and only cross-checked at the beginning and end of the experiment. The score for each animal was calculated by adding the scores assigned to each parameter. It was determined that if the animals’ humane endpoints score reached the critical level (total score of four), they should be re-evaluated and, if necessary, removed from the study and euthanized. Moreover, a score of three in some parameters, like body weight and mental status, was an indicator of euthanasia. Animals were handled regularly before and during the trial to reduce stress during manipulation and avoid any interference with the animal welfare assessment.

When the animals did not clean themselves, a lack of grooming (i.e., the presence of dirt on the hair) was registered. According to Mason et al., chromodacryorrhea can be an indicator of stress and has been described in several studies with laboratory animals [8]. Harderian glands are secretory pigment lacrimal glands located posterior to the ocular globes. These glands release a reddish lipid- and porphyrin-rich material that lubricates the eyes and eyelids [19]. Healthy animals perform grooming frequently, so the deposition of this secretion around the eyes is reduced. However, in discomfort and disease, the animals reduce grooming, and this secretion accumulates around the rat’s eyes and nose. Chromodacryorrhea changes the color of the hair and is also characterized by the presence of excessive reddish secretions [20]. The Grimace scale was used to assess the animals’ pain based on their facial expressions [9]. The animals’ reaction to external stimuli was evaluated by observing their response to hand clapping above their cages. To assess the animals’ hydration status, a skin pinch test was performed. To make this test, the animal was placed on the researcher’s forearm, and the pinch test was made on the back region, more precisely on the lumbar region. If the skin took more than about two seconds to normalize, the rat was considered dehydrated [10]. According to our score sheet, animals with a sum of scores that reach 4 should be sacrificed. However, animals with the following changes, including 20% of body weight loss, severe anemia, stupor and coma, skin necrosis and walking on the tip of the extremities, must be re-evaluated and, if necessary, should be sacrificed. When an animal approaches the critical score level, a veterinarian should conduct a comprehensive health assessment, and if the animal’s condition has deteriorated, it would be necessary to euthanize it.

### 2.6. Body Temperature

Additionally, to the parameters mentioned above, the animals’ body temperature was recorded weekly with a non-contact infrared forehead thermometer (Andon iHealth PT2L, Paris, France). For each animal, the temperature was measured on the forehead, always at the same location, at approximately 3 cm. All readings were performed by the same researcher when the animals stood relaxed in a black-lined cage. To avoid any temperature increase due to stress, all temperature readings were recorded in under one minute.

### 2.7. Blood Glucose Assessed during Protocol

One week after STZ or vehicle administration (3rd week of protocol, see Figure 1), blood glucose level was measured in all animals using a GlucoMen^®^ Areo 2K and blood test strips (A. Menarini Diagnostics, Florence, Italy). The measurements were taken after 12 h of fasting and 2 h after feeding. Blood was taken from the tail by gently “milking” it after a small cut [21]. To promote hemostasis and to perform asepsis at the puncture site, we used a sterile gauze with 3% hydrogen peroxide [22]. A new blood glucose measurement was conducted two weeks later in all animals, after 12 h fasting and 2 h after feeding.

### 2.8. Animals’ Euthanasia and Samples Processing

At the end of week 7, the animals were euthanized, after 12 h fasting, by intraperitoneal administration of ketamine (75 mg/kg, Rompun^®^ 2%, Bayer Healthcare SA, Kiel, Germany) and xylazine (10 mg/kg, Clorketam 1000, Vetoquinol, Barcarena, Oeiras, Portugal), followed by exsanguination by cardiac puncture. Blood samples were collected directly from the heart and centrifuged at 3000 g/10 min for serum separation. Glucose, albumin, triglycerides and cholesterol levels were determined in an autoanalyzer (Prestige 24i, Cornay PZ, Lomianki, Poland). A complete necropsy was performed in all animals, and the internal organs were macroscopically observed, collected, weighed, and preserved in 10% buffered formalin for 24 h for histopathological analysis. A veterinary pathologist blindly examined 4-µm sections of paraffin-embedded kidneys, stained with hematoxylin and eosin (H&E) under a light microscope.

### 2.9. Murinometric and Nutritional Measurements

The animals’ body weight was determined immediately before euthanasia (after 12 h fasting) to determine the Lee index and body weight index (BWI) [23]. After the anesthesia for euthanasia, the abdominal perimeter (AP), the thoracic perimeter (TC) and the nasal-anal length (NAL) of all rats were measured, and the AP/TC ratio was determined [24]. The following formulas were applied:

(3) $$Lee\;index=\sqrt[{3}]{\frac{Final\;body\;weight\;(g)}{NAL\;(cm)}},$$

(4) $$BMI=\frac{Final\;body\;weight\;(g)}{{NAL}^{2}\;({cm}^{2})},$$

Moreover, the specific rate of weight gain (SRWG) and food efficiency coefficient (FEC) were calculated using the weight of the animals before fasting at the end of the experiment:

(5) $$SRWG=\frac{Final\;body\;weight\left(g\right)-Initial\;body\;weight\left(g\right)}{Initial\;body\;weight\left(g\right)\times Days\;of\;the\;experiment},$$

(6) $$FEC=\frac{Final\;body\;weight\left(g\right)-Initial\;body\;weight\left(g\right)}{\text{Animal’s}\;total\;food\;consumption\;(g)},$$

The food consumption of the animals was measured as the mean per cage, and the mean consumption per animal was considered.

### 2.10. Statistical Analysis

All data were analyzed using GraphPad Prism^®^ software for Windows (version 8.0.1, San Diego, CA, USA). The mean and the standard deviation (SD) were calculated for each parameter. Body weight, food and water consumption, glucose levels during the protocol and temperature were compared using the Ordinary two-way ANOVA with Šidák correction for multiple comparisons. For post-mortem glucose, albumin, cholesterol, and triglycerides levels, kidneys’ relative weight, and murinometric and nutritional parameters, the differences between groups were assessed using Student’s t-distribution. Since the values in Table S1, Supplementary Material, do not follow a normal distribution, a non-parametric Mann-Whitney test was performed. *p*-values lower than 0.05 were considered statistically significant. Boxplots were performed to identify outliers. To avoid bias, data analysis was carried out by both researchers related and unrelated to those conducting the animal experiment.

## 3. Results

### 3.1. General Observations

**Figure 2:**
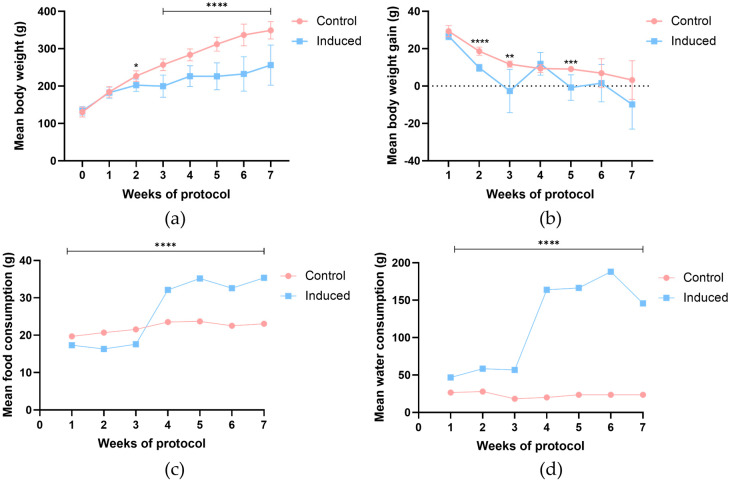
(**a**) Mean body weight of each group, (**b**) mean body weight gain, (**c**) mean food and (**d**) water consumption per day throughout the trial in both experimental groups. * Statistically different from control group (*p* < 0.05); ** *p* < 0.01; *** *p* < 0.001; **** *p* < 0.0001. The group control (n = 8) was intraperitoneally administrated with 0.1 M citrate buffer, while the induced group (n = 16) was intraperitoneally administrated with STZ diluted in 0.1 M citrate buffer after fructose feeding. Data are presented as mean ± standard deviation (SD).

No deaths were recorded during the experimental protocol. The animals’ mean body weight (Figure 2a), after one week of introducing fructose in the animals’ drinking water, was always statistically lower in the induced groups when compared to the controls (week 2, *p* < 0.05; weeks 3–7, *p* < 0.0001). When looking at the body weight gain, some differences were observed between control and induced animals. In fact, a decrease in weight gain over time was observed in both groups, justified by the natural growth of the animals. Throughout the experiment, the weight gain presented several variations in the induced group and was negative in week 3 of the trial (Figure 2b); that was the week after the STZ injection and in the last week. After the STZ injection (week 3), 81.25% of the animals in the induced group showed weight loss. At the end of the protocol, the percentage of animals that lost weight increased to 93.75%. During the consumption of fructose solution, over the first three weeks of the experiment, the animals from the induced group consumed less food when compared with animals from the control group (*p* < 0.0001). However, this was inverted after STZ administration, with the average food consumption in the induced animals higher during the remaining weeks of the experiment (Figure 2c, *p* < 0.0001). Concerning water consumption, induced animals drank more water over time than control animals, and the water intake also increased after STZ injection (Figure 2d, *p* < 0.001).

### 3.2. Animal Welfare

**Figure 3:**
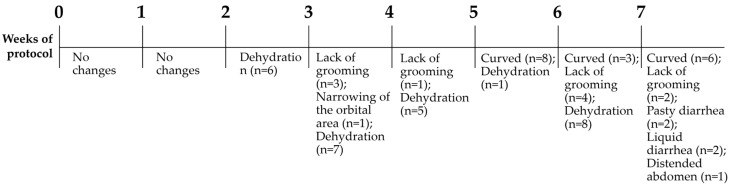
Changes observed in the animals’ welfare over time according to previously established humane endpoints. Animals from the control group did not show any change.

**Figure 4:**
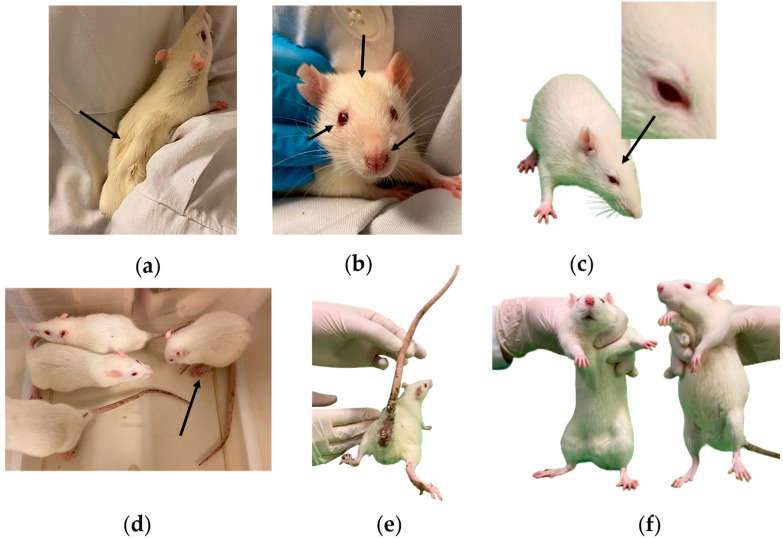
Welfare parameters changed in the induced animals throughout the experimental protocol. (**a**) Dehydration detected by the skin pinch test (black arrow); (**b**) lack of grooming (black arrow); (**c**) narrowing of the orbital area (evaluation of animal expression using the Grimace scale) (black arrow); (**d**) curved posture and isolation from the other animals in the cage (black arrow); (**e**) presence of pasty diarrhea; (**f**) difference between distended abdomen (on the right) and normal abdomen (on the left).

Regarding the assessment of animals’ health status by using the parameters stated in the table of humane endpoints, changes were only observed in the induced group (Figure 3). The first changes were observed in the second week of the experimental protocol, when the animals were being supplemented with fructose solution, with six animals showing dehydration, detected by the skin pinch test as shown in Figure 4a. Changes in animal welfare became increasingly obvious after the STZ administration. Lack of grooming (weeks 3, 4, 6, and 7; Figure 4b), narrowing of the orbital area (week 3; Figure 4c), curved posture and removal of the remaining animals from the cage (weeks 5, 6 and 7; Figure 4d), liquid and pasty diarrhea (week 7; Figure 4e), and distension of the abdomen (Figure 4f) were observed. It is important to note that these changes were not consistent; for example, some animals displayed changes one week, recovered the following week, and the alterations reappeared later. The highest number of alterations in the animals was identified in the last week of the protocol, shortly before they were euthanized. Despite these alterations, none of the animals hit the critical score limit of 4, which implied its removal from the experiment. Nevertheless, the maximum score values assigned to each animal in the induced group were significantly higher when compared to controls (*p* < 0.01; Supplementary Material, Table S1).

### 3.3. Body Temperature

**Figure 5:**
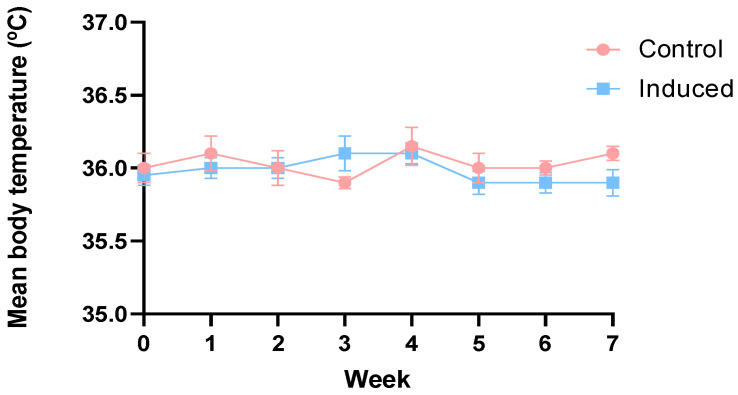
Mean body temperature (°C) recorded weekly per group throughout the protocol. No statistically significant differences were found between the groups (*p* > 0.05). The group control (n = 8) was intraperitoneally administrated with 0.1 M citrate buffer, while the induced group (n = 16) was intraperitoneally administrated with STZ diluted in 0.1 M citrate buffer after fructose feeding. Data are presented as mean ± SD.

No statistically significant variations in the animals’ body temperature were reported during the trial (*p* > 0.05). The mean body temperature of animals from both groups ranged from 35.9 to 36.1 °C (Figure 5).

### 3.4. Kidney Analysis

Animals from the induced group exhibited higher kidneys relative weight (animal kidney weight/animal body weight) when compared to controls, as it is shown in Table 2 (*p* < 0.01, right kidneys; *p* < 0.001, left kidneys).

**Figure 6:**
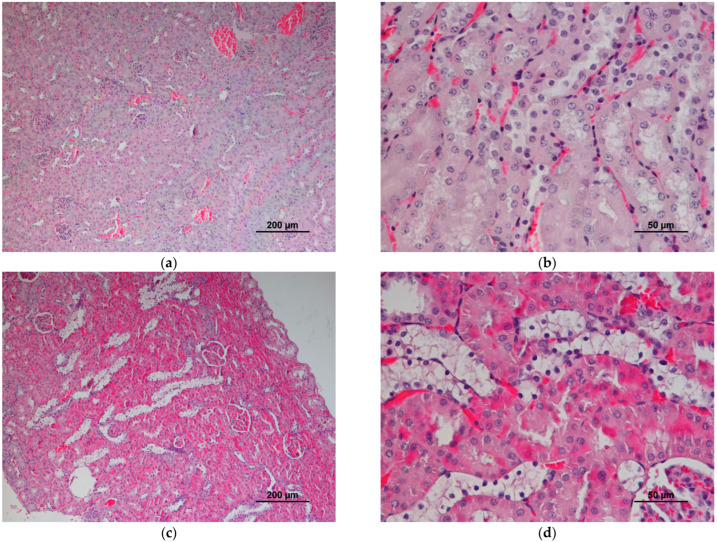
Kidney sections from the animals under study. Images (**a**,**b**) display normal glomeruli and cortical tubules from an animal of the control group. Notice the vacuolation of the cytoplasm and pyknosis of the epithelial cells of cortical tubules. Focal cell necrosis, marked by a darkly stained cytoplasm and sloughed necrotic cells in the lumen of cortical tubules, are also observed in images (**c**,**d**) from fructose-fed and STZ-injected animals.

To gain a better understanding of the increase in fructose-fed and STZ-injected rat kidneys’ relative weights, a histological analysis of this organ was conducted. As illustrated in Figure 6a,b, the histology of the control animals’ kidneys showed normal glomeruli and cortical tubules. However, the histological sections of the induced animals revealed focal cell necrosis, characterized by a darkly stained cytoplasm and sloughed necrotic cells in the lumen of the cortical tubules (Figure 6c,d).

### 3.5. Blood Parameters

When animals presented blood glucose levels higher than the maximum value read by the glucometer (600 mg/dL), the maximum value was attributed to them for the assessment of glycemia in each group. In the first evaluation, 56.25% of the rats exhibited feeding glucose readings higher than 600 mg/dL. This percentage increased to 81.25% in the second measurement (performed in week 6). Three animals from the group induced exhibited blood glucose levels <250 mg/dL 2 h after feeding, which were considered outliers by the statistical analysis performed.

In general, the blood glucose levels were higher after feeding, when compared with those measurements taken after fasting, in both experimental groups, reaching a statistically significant difference in the group induced at the 4th and 6th weeks of the experiment (*p* < 0.0001) (Table 3).

Regarding albumin, total cholesterol, and triglycerides levels, no statistically significant differences were found (*p* > 0.05; Table 4), supporting the absence of systemic inflammation and lipid metabolism alterations, respectively, in fructose-fed and STZ-injected rats. Fasting glucose levels measured in serum samples obtained from the blood samples collected at necropsy were significantly higher in the induced group when compared to the control group (*p* < 0.05; Table 4), suggesting insulin resistance in fructose-fed and STZ-injected rats.

### 3.6. Murinometric and Nutritional Measurements

Except for the initial body weight, the murinometric and nutritional parameters of induced animals were significantly lower when compared with control animals (*p* < 0.01; Table 5). In fact, the trend of the average weight of the induced animals remains the same as the average weight measured during normal feeding of the animals. Mean nasal-anal length, thoracic perimeter, and abdominal perimeter were significantly lower in induced animals (*p* < 0.01) than in control animals, suggesting that the animals’ normal growth was retarded after exposure. The Lee index, BMI, specific rate of body weight gain, and food efficiency coefficient, factors related to an animal’s body weight and food intake, were also significantly lower in chemically induced animals (*p* < 0.001, *p* < 0.01, *p* < 0.0001), respectively), revealing the same tendency as the mean body weight.

## 4. Discussion

In general, and following the EU directive, to perform an experimental protocol in European countries is necessary to request authorization from the institutional authorities in which the experimental procedures will be carried out, as a first phase, followed by an authorization request to the national authority responsible for the supervising of the use of animals for scientific purposes. To avoid animal suffering, the analysis of the humane endpoints is required by both bodies. However, the bibliographic references related to the analysis of the humane endpoints in the most diverse research with laboratory animals are scarce and, according to our experience, often difficult to publish. To overcome this problem, our team has collected as much information as possible on this subject over the years to expand the available knowledge. So, envisioning to contribute to this, this research had as a main goal to provide a list of humane endpoints to the rat model of fructose-fed and STZ-induced type 2 diabetes in male Sprague-Dawley rats, as well as to validate this induction model. STZ, together with fructose, is widely used to induce type 2 diabetes in rats [13].

The choice for a 10% fructose solution was based on a previous study that compared the effects of the administration of 10, 20, 30 and 40% fructose solutions on the development of the disease [13]. Once some animals belonging to the three highest dose groups ended up dying during the trial, to ensure the success of the experiment, the concentration of 10% fructose was chosen. The same study was used as a reference to define the STZ dose. In our study, as the objective was to validate the humane endpoints table, the animals were euthanized after 5 weeks of STZ administration and not after 9 weeks, as reported by Wilson and Islam [13]. Those doses and time points guaranteed diabetes development without compromising severely animals’ welfare. In this study, no deaths were recorded, inversely to that previously described in an animal model of diabetes type 1 induced by the single administration of STZ at 50 mg/kg [25]. The study by Wilson and Islam also reported no animal deaths with the same doses of fructose and streptozotocin [13]. After fructose and STZ administration, the beds and cages of induced animals were changed daily; the animals were handled extensively before and during the experiments. Moreover, the animals were observed daily to check their welfare, available water was verified daily, and scores were attributed to each parameter weekly.

Over the weeks of fructose ingestion, the induced animals consumed more water, probably due to the sweet taste, and ate less food. These animals began to drink and eat more water and food after receiving STZ. Previous research in both male Wistar and Sprague-Dawley rats found similar results [13,26]. Indeed, polydipsia and polyphagia are symptoms of diabetes [27]. Polydipsia (excessive thirst) is commonly linked to high blood glucose levels, as the kidneys produce more urine to eliminate the excess sugar. Consequently, animals tend to drink more water to replace fluid loss [28]. This event can lead to increase kidney damage. Indeed, the kidneys’ relative weight was increased in the induced group when compared to the control group, suggesting that morphological changes may have occurred in these organs. In fact, hypertrophy of cortical tubules due to the vacuolation of the cytoplasm of degenerative epithelial cells was observed in tissue sections of fructose-fed and STZ-injected rats. Polyphagia causes the body to lose energy, which triggers the brain to increase appetite to compensate [29]. Interestingly, Wilson and Islam [13] reported that their type 2 diabetes-induced model did not display polyphagia. According to Chu and collaborators [30], analyzing the circulating levels of leptin is important for validating polyphagia, as this hormone is responsible for balancing food intake and energy expenditure. Polyuria was also observed in the induced rats. This symptom was observed by looking at the bed, soaked in urine and by its smell. It became necessary to change it every day, compared to the weekly bedding change required for the control rat cages. Our results indicate that animals from induced groups showed renal histological changes associated with polyuria and suggest the presence of early signs of diabetes-related nephropathy. In advanced diabetes, the decline of renal function can be attributed to a prolonged state of nitric oxide deficiency that consequently may exacerbate polyuria [31]. In future studies, the urinary levels of albumin should be assessed to better screen the onset of kidney disease. The dehydration observed in the induced animals throughout the weeks was also linked to polyuria and polydipsia [32].

Some altered parameters in the table of humane endpoints, such as lack of grooming, narrowing of the orbital area, and hunched posture, were indicative of animals’ suffering and stress, most likely because of diabetes development. The Grimace scale described in the table was demonstrated as a scale that, analyzing altered facial expressions in rodents, provides a precise and reliable assessment of the animals’ pain status [9]. Throughout our trial, the symptoms related to diabetic neuropathic pain identified by the Grimace scale were identified, including altered eyes position, slight discomfort of the animals during handling and curved posture. Lack of grooming was also analyzed by Yanlin Wang-Fischer and Tina Garyantes in a diabetic rat model as an indicator of disease or stress [33]. Diarrhea, as well as visualization and palpation of the abdomen, were included as typical clinical signs of diabetes mellitus in our humane endpoints table. High blood sugar levels can damage the body’s tiny blood vessels and neurons, resulting in occurrences like the ones described above [34,35]. In fact, in 7th week of protocol, the animals showed a distended abdomen, probably caused by gastrointestinal constipation and the presence of liquid and pasty diarrhea. Other parameters stated in the table remained unchanged throughout the experiment; however, if the protocol were to be extended for a longer duration, there would likely be an increase in both the total score attributed to the animals and several altered parameters, such as body weight loss in fructose-fed and STZ-injected rats. Considering the humane endpoints evaluated, the most useful parameters to assess humane endpoints in this type 2 diabetes induction model were dehydration, absence of grooming, the posture of the animals, abdominal visualization and palpation, and fecal appearance. These results suggest that the parameters used to evaluate animal welfare were sensitive in this model. More than 20 years ago, David Morton proposed a table to determine humane endpoints in an STZ-induced diabetes model in Wistar rats [4]. In that table, each parameter was evaluated as negative or positive and assigned a score. In addition, Morton stated that this assessment should be performed in critical periods, such as after administration and in the middle of the study. Although we did not include parameters such as temperature and food and water consumption in our table of humane endpoints, they were evaluated separately. Effectively, our purpose was to create a table that was relatively simple to evaluate, with detailed information and with a direct score assignment. Moreover, instead of dividing the parameters at a distance and through handling, we used the main parameters, and through the attribution of a score, a brief description of the changes was carried out so that the table can be easily reproducible and the subjectivity reduced. In addition, our observations were recorded weekly, allowing better control of the animal’s health status. A study performed by Wang-Fischer and Garyantes evaluated animal welfare in an animal model of type 1 diabetes. However, the authors only attributed a score to the grooming and the feces appearance. Parameters such as dehydration, sleepiness, unkempt appearance, anemia, skin or eye infection, and morbidity were only subjectively evaluated by a veterinarian, and no score was applied [33]. Furthermore, to the best of our knowledge, no humane endpoint studies have been performed in a rat model of type 2 diabetes with the addition of fructose in drinking water.

We strongly believe that this table will be helpful in future experiments on fructose-fed and STZ-induced type 2 diabetes, and depending on the study’s goals, it can be extended to other animal models of diabetes. To better monitor animal welfare, human endpoints can also include the body temperature of animals [36]. Diabetes is associated with damage to blood vessels and nerves, which can lead to rises in body temperature [37]. However, no differences were seen in the induced animals until the end of the study. Maybe some difference in body temperature between groups may be observed in longer protocols.

The fasting blood glucose levels revealed statistically significant variations at the end of the protocol when the analysis was performed in an automated autoanalyzer, confirming the hyperglycemia associated with diabetes development [38]. Fructose-feeding is known to trigger insulin resistance, islet dysfunction, renal hypertrophy and tubulointerstitial disease, and cataracts, among other complications [39]. In this animal model, serum insulin concentrations were found to be lower in this animal model 9 weeks after STZ administration, which was attributed to fructose-induced oxidative stress and β-cell damage [13]. The same authors observed histological signs of partial β-cell damage and noted sensitivity to anti-diabetic drugs, supporting insulin resistance in this model. Using the same animal model but with access to a diet supplemented with bread, we did not notice significant changes in insulin levels, despite the observed hyperglycemia, 5 weeks after STZ injection. Our data suggest that at this stage of the disease, insulin is not able to stimulate glucose uptake in striated muscles and adipose tissues [40]. As the disease progresses, islet cells became severely damaged and unable to secrete insulin [13]. At the end of the experiment, the induced animals (fasted or not) had a lower mean body weight compared to the controls. These findings are in accordance with previous studies in this diabetes induction model [33,41]. Effectively, these results are not related to food and water consumption, as the induced animals had a higher consumption, but they may be associated with increased energy expenditure [42]. The induced groups had lower Lee’s index, BMI, and specific rate of body weight gain when compared with control animals. The feed efficiency coefficient is a scale that represents the conversion of the consumed food into animals’ weight gain [23]. The lower values of this variable can be explained by the higher food consumption of induced animals compared to controls. This result can be explained by the typical weight loss seen in diabetic rats, as stated before, as well as the polyphagia phenomena.

## 5. Conclusions

Our results suggest that our table of humane endpoints is appropriate to monitor the animals’ welfare in this rat model of type 2 diabetes STZ-induced following fructose ingestion and can be used by other researchers using this model and eventually expanded to other models of diabetes to successfully ensure animals’ welfare. In this study, none of the biological parameters appeared to justify the premature sacrifice of any of the animals; however, if the sum of the assigned scores is equal to or higher than four, the animal must be carefully evaluated and considered its sacrifice. 

## Figures and Tables

**Table 1:** Humane endpoints applied to the rat model of chemically induced type 2 diabetes. Adapted from Silva-Reis et al., Faustino-Rocha et al. and CCAC guidelines [3,10,17].

Score		0	1	2	3
Body Condition/Weight		Normal	Altered body condition/weight loss of <10%	Emaciated/weight loss of 10–20%	Weight loss of >20% (Euthanasia)
Posture		Normal	Curved	---	---
Hair/tail appearance and grooming		Normal	Lack of grooming	Very bad-looking hair, dirty tail, severe chromodacryorrhea	---
Grimace scale	Eyes and extremities	Normal	Narrowing of the orbital area	Moderate anemia/Cataracts, Eyes fully closed	Severe anemia/Corneal ulcers (Euthanasia)
	Position of ears and whiskers	Normal	Dropped ears, forward whiskers (Stiffened)	Floppy and curved ears, whiskers turned forward and crumpled (clusters)	---
	Nose/cheeks	Normal	Flattening and elongating the tip of the nose	Flattening of the cheeks (cheek appearance)	---
Walk		Normal	---	---	Walking on the tip of the extremities (Euthanasia)
Skin		Absence	Skin injuries/infections <5 mm	Skin injuries/infections >5 mm	Presence of necrosis (Euthanasia)
Mental status		Normal	Inactive	Moribund	Stupor/coma (Euthanasia)
Response to external stimuli		Normal	Moderate	Moderate with vocalization	Violent
Hydration status		Normal	Abnormal skin pinch test (>2 s)	---	---
Stool appearance		Solid	Pasty diarrhea	Liquid diarrhea	---
Convulsions		Absence	---	---	Presence
Visualization and response to mild abdominal palpation		Normal	Vocalization	Distended	---

Recommendation: A total score of 4 or a score of 3 in some parameters, like body weight, were indicators for euthanasia. When an animal’s score exceeds a critical level, it was re-evaluated to decide if it should be removed from the protocol.

**Table 2:** Mean relative weight of rats’ kidneys after euthanasia.

Relative Weight (g/kg)	Control (n = 6)	Induced (n = 16)
Right kidney	4.053 ± 0.165	4.952 ± 0.676 ^a^
Left kidney	3.861 ± 0.266	4.802 ± 0.593 ^b^

^a^ Statistically different from the control group (*p* < 0.01), ^b^ *p* < 0.001. Data are presented as mean ± SD. The group control was intraperitoneally administrated with 0.1 M citrate buffer, while the induced group was intraperitoneally administrated with STZ diluted in 0.1 M citrate buffer after fructose feeding.

**Table 3:** Mean blood glucose levels per group after 12 h fasting and 2 h after feeding in both experimental groups at the 4th and 6th weeks of the experiment.

		Glucose Levels (mg/dL)	
Week of Protocol	Timepoint	Control (n = 8)	Induced (n = 16)
4	After fasting (12 h)	67.00 ± 8.97	65.06 ± 19.06
	After feeding (2 h)	141.00 ± 15.22	568.92 ± 71.43 ^a^
6	After fasting (12 h)	94.63 ± 8.35	110.63 ± 30.25
	After feeding (2 h)	141.63 ± 9.55	600.00 ± 0.00 ^a^

^a^ Statistically different from the control group (*p* < 0.0001). Data are presented as mean ± SD. The group control was intraperitoneally administrated with 0.1 M citrate buffer, while the induced group was intraperitoneally administrated with STZ + 0.1 M citrate buffer.

**Table 4:** Albumin, total cholesterol, glucose, and triglycerides serum levels per group after animals’ euthanasia.

	Control (n = 6)	Induced (n = 16)
Albumin (g/dL)	2.28 ± 0.66	1.81 ± 0.84
Cholesterol (mg/dL)	47.75 ± 10.24	52.19 ± 21.17
Glucose (mg/dL)	107.87 ± 25.23	251.73 ± 136.84 ^a^
Triglycerides (mg/dL)	36.77 ± 12.88	60.52 ± 45.23

^a^ Statistically different from the control group (*p* < 0.05). The group control was intraperitoneally administrated with 0.1 M citrate buffer, while the induced group was intraperitoneally administrated with STZ diluted in 0.1 M citrate buffer after fructose feeding.

**Table 5:** Murinometric and nutritional parameters of animals from both groups control and induced.

Parameter	Control (n = 8)	Induced (n = 16)
Initial body weight (g)	130.57 ± 12.29	133.75 ± 10.92
Final body weight (g)	349.33 ± 21.76	256.07 ± 51.99 ^c^
Final fasting body weight (g)	328.75 ± 18.94	235.01 ± 51.41 ^c^
Nasal-anal length (cm)	23.50 ± 0.79	21.03 ± 1.39 ^b^
Thoracic perimeter (cm)	15.13 ± 0.60	13.22 ± 1.22 ^b^
Abdominal perimeter (cm)	15.88 ± 0.70	14.38 ± 1.08 ^a^
Lee index	2.41 ± 0.04	2.22 ± 0.11 ^b^
BMI (g/cm^2^)	0.60 ± 0.03	0.53 ± 0.06 ^a^
Specific rate of body weight gain (g/g)	0.03 ± 0.00	0.02 ± 0.0 ^c^
Food efficiency coefficient (g/g)	1.41 ± 0.14	0.66 ± 0.28 ^c^

^a^ Statistically different from group control (*p* < 0.01); ^b^ *p* < 0.001; ^c^ *p* < 0.0001. The group control was intraperitoneally administrated with 0.1 M citrate buffer, while the induced group was intraperitoneally administrated with STZ diluted in 0.1 M citrate buffer after fructose feeding. Data are presented as mean ± SD.

## Data Availability

The data that support the findings of this study are available on request from the corresponding author.

## Supplementary Materials

The following supporting information can be downloaded at: https://www.mdpi.com/article/10.3390/ani13081397/s1, Table S1: The final score assigned to each animal throughout the trial.
