# Supplementary material for: Studying and Analyzing Humane Endpoints in the Fructose-Fed and Streptozotocin-Injected Rat Model of Diabetes
Source: Animals (Basel). 2023 Apr 18;13(8):1397. doi: 10.3390/ani13081397 (PMC10135389; doi:10.3390/ani13081397)
Supplement: Supplementary file 1 [file animals-13-01397-s001.zip › animals-2267356-supplementary.pdf]

animals

Table S1. The final score assigned to each animal throughout the trial.

	Weeks	0	1	2	3	4	5	6	7	Maximum score assigned
Control (n=8)	1	0	0	0	0	0	0	0	0	0
	2	0	0	0	0	0	0	0	0	0
	3	0	0	0	0	0	0	0	0	0
	4	0	0	0	0	0	0	0	0	0
	5	0	0	0	0	0	0	0	0	0
	6	0	0	0	0	0	0	0	0	0
	7	0	0	0	0	0	0	0	0	0
	8	0	0	0	0	0	0	0	0	0
Induced (n=16)	1	0	0	0	0	0	0	1	1	1
	2	0	0	0	2	1	1	1	1	2
	3	0	0	1	1	1	1	2	1	2
	4	0	0	0	1	1	1	1	3	3
	5	0	0	1	0	0	0	1	3	3
	6	0	0	0	1	0	0	0	0	1
	7	0	0	1	1	0	0	1	0	1
	8	0	0	0	0	0	1	0	0	1
	9	0	0	1	1	1	1	1	3	3
	10	0	0	0	0	0	0	1	0	1
	11	0	0	0	0	0	1	1	2	2
	12	0	0	0	2	0	1	1	2	2
	13	0	0	1	0	0	0	1	1	1
	14	0	0	0	2	0	0	1	3	3
	15	0	0	0	0	2	1	2	1	2
	16	0	0	1	0	0	0	0	0	1

The group control (n = 8) was intraperitoneally administrated with 0.1 M citrate buffer, while the induced group (n = 16) was intraperitoneally administrated with STZ + 0.1 M citrate buffer.
